# Defining the impact of melanopsin missense polymorphisms using *in vivo* functional rescue

**DOI:** 10.1093/hmg/ddy150

**Published:** 2018-04-30

**Authors:** Jessica Rodgers, Steven Hughes, Carina A Pothecary, Laurence A Brown, Doron G Hickey, Stuart N Peirson, Mark W Hankins

**Affiliations:** 1 Nuffield Department of Clinical Neurosciences, Sleep and Circadian Neuroscience Institute, OMPI G, Sir William Dunn School of Pathology, University of Oxford, Oxford, UK; 2 Nuffield Laboratory of Ophthalmology, Nuffield Department of Clinical Neurosciences, West Wing, John Radcliffe Hospital, University of Oxford, Oxford, UK

## Abstract

Melanopsin (OPN4) is an opsin photopigment expressed within intrinsically photosensitive retinal ganglion cells (ipRGCs) that mediate non-image forming (NIF) responses to light. Two single-nucleotide polymorphisms (SNPs) in human melanopsin (*hOPN4*), *Pro10Leu* and *Thr394Ile*, have recently been associated with abnormal NIF responses to light, including seasonal affective disorder. It has been suggested these behavioural changes are due to altered melanopsin signalling. However, there is currently no direct evidence to support this. Here we have used ipRGC-specific delivery of *hOPN4* wild-type (*WT*), *Pro10Leu* or *Thr394Ile* adeno-associated viruses (AAV) to determine the functional consequences of *hOPN4* SNPs on melanopsin-driven light responses and associated behaviours. Immunohistochemistry confirmed *hOPN4* AAVs exclusively transduced mouse ipRGCs. Behavioural phenotyping performed before and after AAV injection demonstrated that both *hOPN4 Pro10Leu* and *Thr394Ile* could functionally rescue pupillary light responses and circadian photoentrainment in *Opn4^−/−^* mice, with no differences in NIF behaviours detected for animals expressing either SNP compared to *hOPN4 WT*. Multi-electrode array recordings revealed that ipRGCs expressing *hOPN4 Thr394Ile* exhibit melanopsin-driven light responses with significantly attenuated response amplitude, decreased sensitivity and faster offset kinetics compared to *hOPN4 WT*. IpRGCs expressing *hOpn4 Pro10Leu* also showed reduced response amplitude. Collectively these data suggest *Thr394Ile* and *Pro10Leu* may be functionally significant SNPs, which result in altered melanopsin signalling. To our knowledge, this study provides the first direct evidence for the effects of *hOPN4* polymorphisms on melanopsin-driven light responses and NIF behaviours *in vivo*, providing further insight into the role of these SNPs in melanopsin function and human physiology.

## Introduction

Melanopsin is an opsin photopigment expressed in a subset of retinal ganglion cells in the mammalian retina. These intrinsically photosensitive retinal ganglion cells (ipRGCs) mediate a wide range of non-image forming (NIF) responses to light, including circadian photoentrainment (1–2), pupillary light responses (PLR) (3) and period lengthening under constant light (2,4). In addition to endogenous melanopsin-driven photosensitivity, ipRGCs also receive input from rods and cones (5), and thus ipRGC output represents a complex interaction between rod, cone and melanopsin-driven responses (6).

To date, two single-nucleotide polymorphisms (SNPs) in the human melanopsin (*hOPN4*) gene have been associated with abnormal NIF behaviour: *Pro10Leu* (rs2675703, NM_033282.3: c.29C>T) and *Thr394Ile* (rs1079610, NM_033282.3: c.1181C>T). For *Pro10Leu*, Roecklein *et al.* (7) found a higher frequency of the TT genotype (leucine at amino acid position 10) in individuals with seasonal affective disorder (SAD), a mood disorder where decreased daylength is accompanied by increased frequency of depressive episodes (8). Individuals with the TT genotype also demonstrated greater susceptibility to changes in daylength on their sleep onset time and chronotype (9), not seen for the CT and CC genotypes. The impact of *Thr394Ile* on melanopsin-associated behaviour has also been explored. Individuals with the TT genotype (isoleucine at position 394) reportedly display attenuated PLR to white light (10) and high intensity monochromatic blue, but not red, light (11,12). These pupil deficits were not seen in individuals with the CT and CC genotypes. Lee *et al.* (13) also found that individuals with the *Thr394Ile* CC genotype had disrupted sleep-wake timing, compared to CT or TT genotypes.

These association studies of naturally occurring *hOPN4* polymorphisms have suggested that variation in the melanopsin gene provides an underlying mechanism for abnormal NIF behaviour. However, such genetic association-based approaches do not establish a causal link between presence of the *hOPN4* SNP and disruption of melanopsin-driven photic responses. To fully understand the impact of these SNPs on human physiology, it is essential to establish a direct connection between the missense polymorphisms and altered melanopsin function.

*Pro10Leu* and *Thr394Ile* are located in the N and C terminus of the melanopsin protein, respectively. Based on amino acid alignment of melanopsin from 15 species (see Materials and Methods section for details), these domains are not strongly conserved between melanopsins of different species and seem to be grouped along phylogenetic lines. For example, mice possess *Leu10*, while primates possess *Pro10*. Similarly, *Thr394* seems to be conserved in mammals, while non-mammals possess *Ser394*. While it is possible this variation reflects biological differences, it can also suggest that specific amino acids at these sites are not integral to the structure or function of melanopsin protein. Furthermore, mutation of the equivalent site to *hOPN4 Thr394Ile* in mouse melanopsin does not change the kinetics of intracellular calcium responses following heterologous expression in Hek293T cells (14). Thus, there is limited evidence currently available from bioinformatics and *in vitro* studies to support the hypothesis that *Pro10Leu* and *Thr394Ile* significantly affect the function of the melanopsin protein. However, it is likely that *in vitro* cell line expression systems fail to mimic the cellular environment of ipRGCs and do not fully replicate all aspects of the melanopsin signalling cascade, potentially restricting the usefulness of these approaches when studying the effects of *hOPN4* SNPs.

To determine the functional consequences of *hOPN4* SNPs on melanopsin function and associated NIF behaviours, we have employed a novel approach using conditionally targeted adeno-associated viruses (AAV) to introduce either *hOPN4* wild-type (WT), *hOPN4 Pro10Leu* or *hOPN4 Thr394Ile* SNP variants specifically to ipRGCs of mice lacking endogenous melanopsin expression (*Opn4*^*−*^^*/*^^*−*^). This model allows the assessment of *hOPN4*-driven behaviours and ipRGC light responses under native cellular conditions. Our data show that both *hOPN4 Pro10Leu* and *Thr394Ile* fully restored PLR and circadian photoentrainment to *Opn4*^*−*^^*/*^^*−*^ mice, with no differences in NIF behaviours detected for animals expressing either SNP compared to *hOPN4 WT* controls. However, multi-electrode array (MEA) recordings demonstrate that ipRGCs expressing *hOPN4 Thr394Ile*, but not *Pro10Leu*, exhibit melanopsin-driven light responses with altered sensitivity and kinetics compared to *hOPN4 WT* controls. Both *Pro10Leu* and *Thr394Ile* also result in attenuated response amplitude, suggesting that these SNPs may directly impact melanopsin function.

## Results

### ipRGC-specific expression of *hOPN4* in *Opn4*^*−*^^*/*^^*−*^*Cre^+/+^* mice

To generate an animal model of *hOPN4* SNPs, AAVs containing a double-floxed inverted orientation (DIO) *hOPN4* transgene expression cassette were injected into the vitreous of melanopsin deficient *Opn4*^*−*^^*/*^^*−*^*Cre^+/+^ eYFP* mice to facilitate ipRGC-specific *hOPN4* expression *in vivo*. This melanopsin ‘replacement’ technique was first validated to confirm it was suitable for assessing the impact of *hOPN4* SNPs on melanopsin function and whole animal physiology.

**Figure 1.**
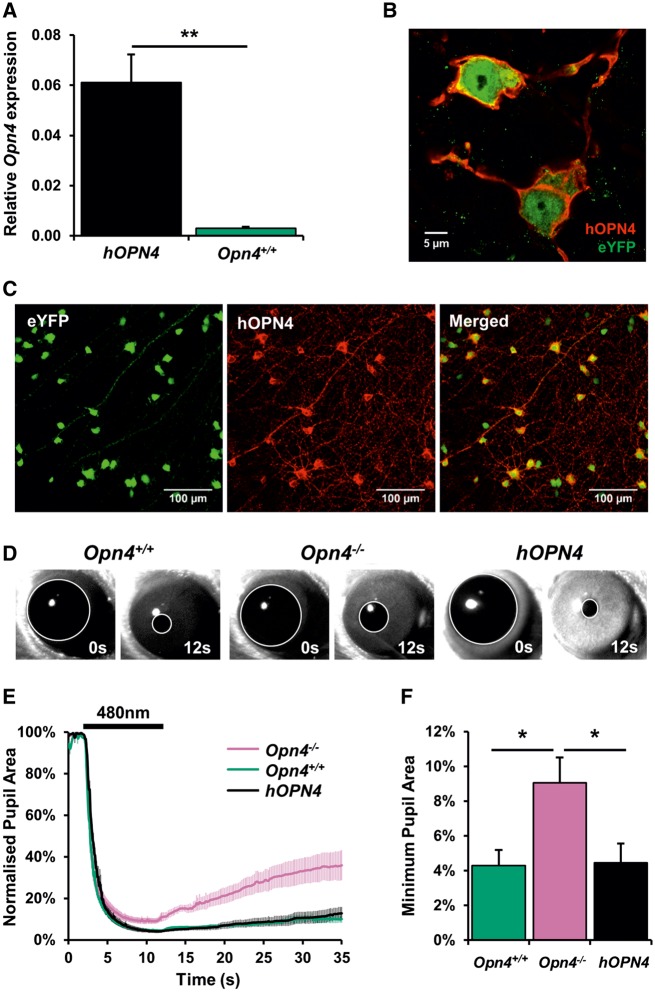
Targeted *hOPN4* AAV delivery restores ipRGC-specific melanopsin expression and wildtype pupil phenotype to *Opn4^−/−^ Cre^+/+^* mice. (A) Relative mRNA expression of melanopsin in *Opn4^+/+^* animals and *Opn4^−/−^ Cre^+/+^* treated with *hOPN4* AAV (*hOPN4*), assessed using primers that bind to an identical sequence in mouse and human melanopsin. Expression data are shown normalized to the geometric mean expression of 4 housekeeping genes (*Arbp, Psmb2, Gapdh* and *B2m*), *n* = 5–7 retina for each group. ** *P* < 0.01 Student’s *t*-test. (B, C) Immunostaining shows co-localization of AAV-delivered hOPN4 (red) and eYFP (green) in ipRGCs of *Opn4^−/−^ Cre^+/+^ eYFP^+/+^* retina. (**D**) Representative images of pupil before (0 s) and at end of light pulse (12 s) (**E**) Kinetics of average pupil light response to a 10 s light pulse (480 ± 10 nm, 14.7 log quanta, shown as black bar above traces) from *Opn4^−/−^ Cre^+/+^* mice treated with *hOPN4 WT* AAV (hOPN4 WT), *Opn4^−/−^* and *Opn4^+/+^* animals, *n* = 6 animals in each group. (**F**) Normalized minimum pupil area at end of light exposure. * *P* < 0.05, Tukey’s HSD *post-hoc* test. Data are shown ± S.E.M. Error bars smaller than symbols are not shown.

Successful transduction of ipRGCs within the mouse retina was determined by expression of AAV-delivered *hOPN4* in *Opn4*^*−*^^*/*^^*−*^*Cre^+/+^ eYFP* mice (Fig. 1), which possess Cre recombinase and eYFP in all subtypes of ipRGCs (15,16). Immunostaining of flatmounted retinas from *hOPN4* AAV-treated *Opn4*^*−*^^*/*^^*−*^*Cre^+/+^* mice confirmed that expression of *hOPN4* was restricted to ipRGCs, with anti-hOPN4 staining exclusively observed in eYFP positive ipRGCs (Fig. 1B and C). Anti-hOPN4 labelling was observed in the plasma membrane and along cellular processes of ipRGCs, suggesting AAV-delivered hOPN4 was successfully trafficked in these cells. Anti-hOPN4 labelling was not observed in the retinas of uninjected *Opn4*^*−*^^*/*^^*−*^*Cre^+/+^* mice (Supplementary Material, Fig. S1A).

Levels of hOPN4 expression were dependent on viral titre (Supplementary Material, Fig. S1B). At the highest viral titre (2.2 × 10^12^ viral genomes/ml or vg/ml), the distribution of hOPN4 expression was found to be widespread throughout the retina, with a high proportion of eYFP-labelled ipRGCs co-labelled for anti-hOPN4 (typically ∼70%). Following *hOPN4* AAV treatment similar levels of hOPN4 expression were observed in all subtypes of ipRGC, with levels of melanopsin expression in non-M1 ipRGCs (and most notably M4 and M5 type ipRGCs) detected at higher levels compared to native *Opn4^+/+^* retina (15–17). RT-qPCR analysis confirmed an overall increase in levels of *hOPN4* mRNA expression in *Opn4*^*−*^^*/*^^*−*^*Cre^+/+^*retina compared to levels of endogenous *Opn4* mRNA expressed in *Opn4^+/+^*retinas (Fig. 1A)*.* Using primers that amplified conserved regions of human and mouse melanopsin, *hOPN4* was found to be expressed at significantly elevated levels (∼×40-fold increase) compared to endogenous melanopsin from *Opn4^+/+^*retinas, *t*(6.08) = 5.18, *P* = 0.002 (Student’s *t*-test, equal variance not assumed).

### ipRGC-specific expression of *hOPN4* rescues the pupil light response of *Opn4*^*−*^^*/*^^*−*^*Cre^+/+^* mice

We next confirmed whether AAV-delivered *hOPN4* was able to rescue the PLR phenotype of *Opn4*^*−*^^*/*^^*−*^*Cre^+/+^* mice. Prior to AAV treatment, *Opn4*^*−*^^*/*^^*−*^*Cre^+/+^* mice showed a characteristic *Opn4*^*−*^^*/*^^*−*^ phenotype with a failure to reach maximal pupil constriction under bright light (3). One month following AAV injection, the PLR of *hOPN4* AAV-treated *Opn4*^*−*^^*/*^^*−*^*Cre^+/+^* mice was comparable with *Opn4^+/+^*mice, with AAV-injected mice exhibiting constriction to ∼5% of dark-adapted pupil size in response to a bright light stimulus (10 s, 480 nm, 14.7 log quanta). By comparison, untreated *Opn4*^*−*^^*/*^^*−*^ mice showed attenuated constriction to bright light, reaching ∼10% dark-adapted pupil size (Fig. 1D and E). Minimum pupil area was significantly smaller in *Opn4^+/+^* (*P* = 0.030, Tukey’s HSD) and *hOPN4* AAV-treated *Opn4*^*−*^^*/*^^*−*^*Cre^+/+^*mice (*P* = 0.037, Tukey’s HSD) compared to untreated *Opn4*^*−*^^*/*^^*−*^ mice (Fig. 1F), *F*(2, 15) = 5.258, *P* = 0.019 (one-way ANOVA), confirming that the PLR phenotype of *Opn4*^*−*^^*/*^^*−*^*Cre^+/+^*mice was functionally rescued by *hOPN4 WT* AAV delivery. Viral restoration of the PLR phenotype was found to be dose-dependent, with higher viral concentrations leading to increased pupil constriction (Supplementary Material, Fig. S1C and D). Complete restoration of the PLR was observed only at the highest titre tested, 10^12^ vg/ml, whereas 10^11^ vg/ml resulted in only partial restoration and the lowest titre tested, 10^10^ vg/ml, had no effect on the PLR. These observations correlate with levels of hOPN4 expression detected using immunohistochemistry following treatment with differing titres of *hOPN4* AAV (Supplementary Material, Fig. S1B).

### Pupil light responses are comparable between *hOPN4 WT* and SNPs, *Pro10Leu* or *Thr394Ile*

Following the validation of ipRGC-specific delivery and functional restoration of melanopsin-dependent behaviours by *hOPN4 WT* AAV treatment, we next examined the impact of *hOPN4* SNPs, *Pro10Leu* and *Thr394Ile* on a range of NIF responses to light, including PLR and circadian entrainment. The principal comparison of interest was whether the *hOPN4 SNP* AAVs demonstrated observable differences in their activity or influence on behaviour relative to the *hOPN4 WT* AAV. Any disruption of melanopsin activity caused by introduction of SNPs *Pro10Leu* and *Thr394Ile* would be expected to result in reduced restoration of NIF responses compared to *hOPN4 WT* controls.

**Figure 2.**
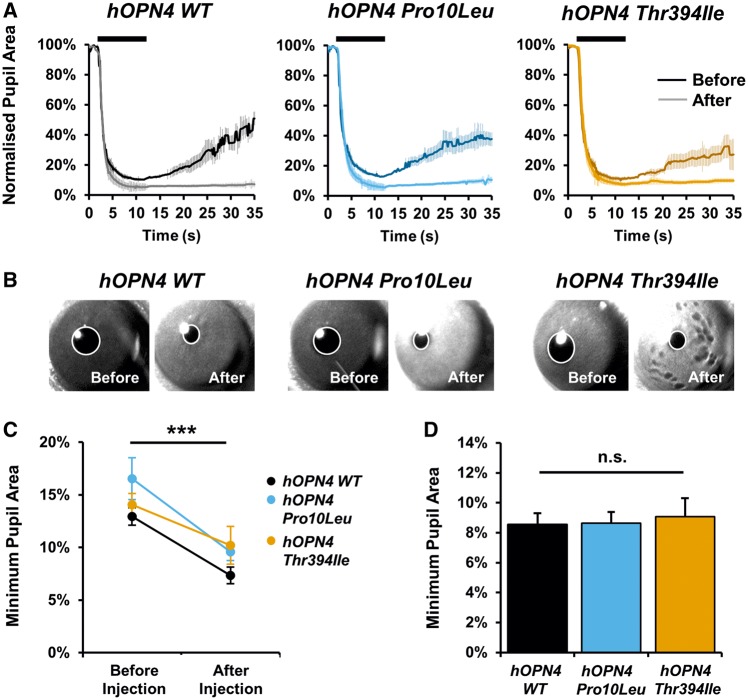
Pupil light responses (PLR) are comparable between *Opn4^−/−^ Cre^+/+^* mice treated with either *hOPN4 Pro10Leu*, *Thr394Ile* or *hOPN4 WT* control AAV. (**A**) Kinetics of average representative PLR to 10 s light pulse (480 nm, 14.7 log quanta, shown as black bar) from the same *Opn4^−/−^ Cre^+/+^*mice before (dark coloured line) and 1 month after (light coloured line) injection of *hOPN4 WT, Pro10Leu* or *Thr394Ile* AAV. (**B**) Representative images of maximum pupil constriction at end of 10 s 480 nm light from the same mice before and after injection. (**C**) Restoration of pupil constriction is comparable between all three *hOPN4* groups, *n* = 3–4 animals in each group, ** *P* < 0.01 main effect of injection, two-way mixed ANOVA. (D) Pupil constriction after injection is comparable in *hOPN4 WT, Pro10Leu* and *Thr394Ile* AAV-treated mice, *n* = 5–8 for each group. n.s. = not significant, one-way ANOVA. Data are shown ± S.E.M. Error bars smaller than symbols are not shown.

The first NIF behaviour tested was the PLR. Again, prior to AAV treatment, all *Opn4*^*−*^^*/*^^*−*^*Cre^+/+^* mice displayed a characteristic *Opn4*^*−*^^*/*^^*−*^ phenotype with a failure to reach maximal pupil constriction following bright light stimulation. When the same *Opn4*^*−*^^*/*^^*−*^*Cre^+/+^*mice were tested after injection of either *hOPN4 WT, Pro10Leu* or *Thr394 Ile* AAV, we observed increased pupil constriction in response to bright light (10 s, 480 nm, 14.7 log quanta, Fig. 2A and B) compared to values observed before injection. Minimum pupil size was consistently smaller after injection for all three viruses, *F*(1, 7) = 114.57, *P* < 0.001, with no interaction of virus by injection, *F*(2, 7) = 2.76, *P* =0.131 (two-way mixed ANOVA, Fig. 2C). However, no differences in maximum pupil constriction were observed between *Opn4*^*−*^^*/*^^*−*^*Cre^+/+^*mice injected with *hOPN4 WT* or either *hOPN4* SNP (Fig. 2D), *F*(2, 14) = 0.09, *P* = 0.917 (one-way between subjects ANOVA).

We also examined pupil constriction using a sub-saturating light stimulus at a longer wavelength (600 nm, 14.7 log quanta, Supplementary Material, Fig. S2). Based on the peak spectral sensitivity of melanopsin at ∼479 nm (18), the 600 nm stimulus represents a ∼2.5 log decrease in light intensity relative to 480 nm and, as anticipated, attenuated pupil constriction was observed in all three virus conditions for the 600 nm stimulus compared to 480 nm (Supplementary Material, Fig. S2A and B). No difference in maximum constriction was found between *hOPN4 WT* and *hOPN4* SNPs when tested using the 600 nm stimulus (Supplementary Material, Fig. S2C). We did observe a trend toward faster pupil recovery in animals injected with *hOPN4 Thr394Ile* compared to *WT* after exposure to 600 nm, but not 480 nm light (Supplementary Material, Fig. S2D). No difference was observed between *hOPN4 P10L* and *WT* control at either wavelength. Interpretation of differences in pupil recovery is complicated as fully sustained pupil constriction after light offset can also be detected in *Opn4*^*−*^^*/*^^*−*^ mice, suggesting this component of the PLR may not be entirely melanopsin-dependent in mice.

Overall, our pupillometry data suggest the introduction of *hOPN4* SNPs does not lead to an observable change in a known melanopsin-driven pupil phenotype, with both *hOPN4 Pro10Leu* and *Thr394Ile* capable of rescuing *Opn4*^*−*^^*/*^^*−*^ maximum pupil constriction to similar levels as *hOPN4 WT* controls.

### Light-induced phase delays are comparable between *hOPN4 WT*, *Pro10Leu* and *Thr394Ile*

Next, circadian photoentrainment of *hOPN4* AAV-treated *Opn4*^*−*^^*/*^^*−*^*Cre^+/+^*mice was examined. It has previously been suggested these behaviours may be disrupted in individuals with either *Pro10Leu* or *Thr394Ile* (9,13). To test this association, a circadian screen was conducted using passive infra-red (PIR) detection to monitor the locomotor activity of *Opn4*^*−*^^*/*^^*−*^*Cre^+/+^*mice injected with hOPN4 *WT*, *Pro10Leu* and *Thr394Ile* AAV. All animals entrained to a 12: 12 light: dark (LD) cycle and exhibited similar period in constant dark before and after injection, as anticipated given that these behaviours are unaffected by genetic ablation of melanopsin (2,4,19). To assess melanopsin-driven circadian behaviour, we measured the phase delay induced by a 15 min 100 lux white light pulse at ZT15, a response that is characteristically attenuated in *Opn4*^*−*^^*/*^^*−*^ mice (2,20). Any disruption of melanopsin activity caused by missense SNPs would be expected to produce smaller phase shifts compared to *hOPN4 WT* controls.

**Figure 3.**
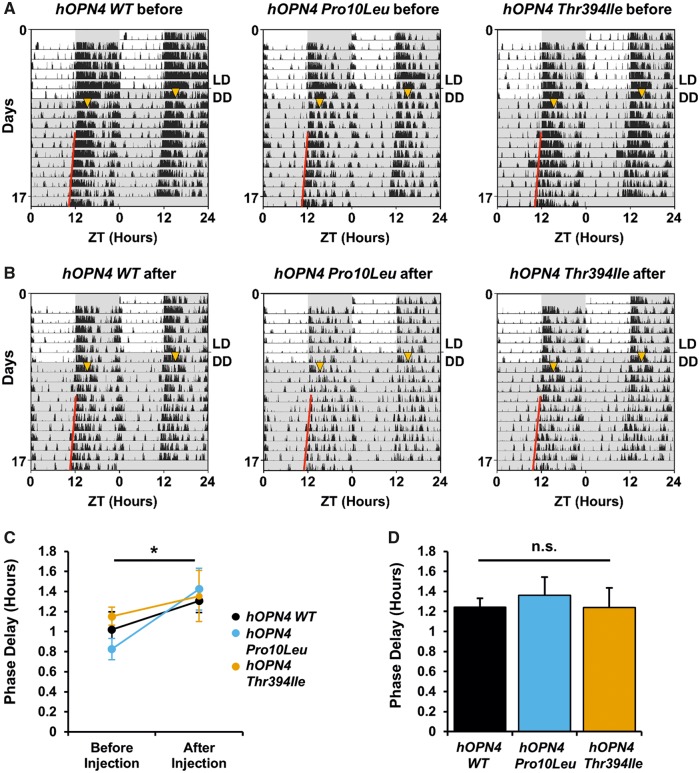
Phase delays are comparable between *Opn4^−/−^ Cre^+/+^* mice treated with either *hOPN4 Pro10Leu*, *Thr394Ile* or *hOPN4 WT* control AAV. (**A**, **B**) Representative actograms showing activity recorded by PIR detection from three *Opn4^−/−^ Cre^+/+^* mice tested (A) before and (B) after bilateral intraocular injection of *hOPN4 WT* (left), *Pro10Leu* (middle) and *Thr394Ile* (right) AAV. Animals were in 12: 12 LD cycle under white light (∼350 lux) for 6 days, then released into constant dark (DD) for up to 10 days. On the first day of DD animals were given a 15 min 100 lux white light pulse at ZT15 (shown as yellow triangle). Actograms are double plotted on a 24 h timescale. Grey shading indicates periods of darkness. Regression line (red) was used to calculate phase delay from onset of activity on first day of DD. Activity data are displayed in 1 min bins. (**C**) Increase in phase delay after injection is comparable between *hOPN4 WT*, *Pro10Leu* and *Thr394Ile*. *n* = 4–5 for each group test. * *P* < 0.05 main effect of injection, two-way mixed ANOVA. (**D**) Phase delays are similar in all three *hOPN4* AAV conditions. *n* = 6–7 for each group, n.s. = not significant, one-way ANOVA. Data are shown ± S.E.M.

Although phase delays were elicited both pre and post-injection (Fig. 3A and B), the magnitude of this delay was significantly increased following AAV treatment, *F*(1, 11) = 7.77, *P* = 0.018 (Fig. 3C). There was no main effect of virus, *F*(2, 11) = 0.26, *P* = 0.774, or interaction of virus with injection, *F*(2, 11) = 0.86, *P* = 0.451 (two-way mixed ANOVA), suggesting the increase in phase shift after injection was similar for *hOPN4 WT*, *Pro10Leu* and *Thr394Ile* AAV. No differences in phase delay were observed between *Opn4*^*−*^^*/*^^*−*^*Cre^+/+^* mice injected with *hOPN4 WT* AAV or either *hOPN4* SNP, *F*(2, 21) = 0.17, *P* = 0.847 (one-way ANOVA, Fig. 3D). These data corroborate our findings from the PLR experiments showing SNPs do not affect melanopsin protein function sufficiently to cause an observable change in *hOPN4*-driven behaviours.

### Period lengthening is comparable between *hOPN4 WT*, and SNPs, *Pro10Leu* or *Thr394Ile*

**Figure 4.**
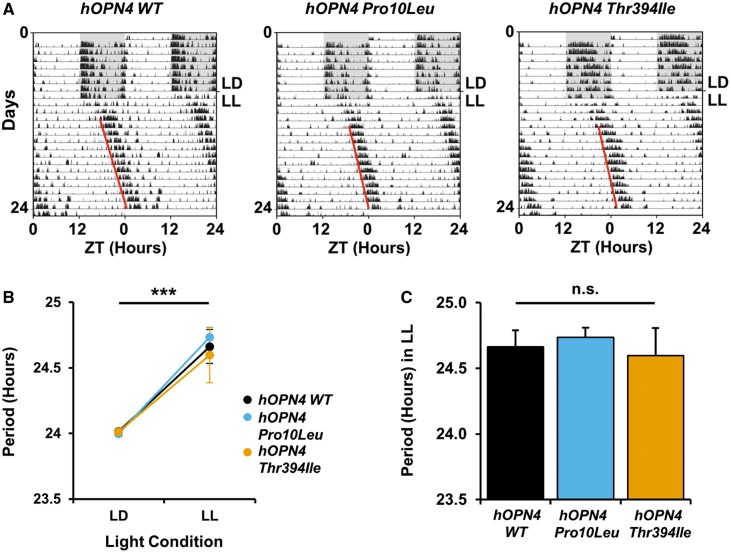
Period lengthening in constant light is comparable between *Opn4^−/−^ Cre^+/+^* mice treated with *hOPN4 Pro10Leu*, *Thr394Ile* and *hOPN4 WT* control AAV. (**A**) Representative actograms showing activity recorded by PIR detection from three *Opn4^−/−^ Cre^+/+^* mice after bilateral intraocular injection of *hOPN4 WT* (left), *Pro10Leu* (middle) and *Thr394Ile* AAV (right). Animals were in 12: 12 LD cycle under white light (∼350 lux) for 8 days, then released into constant white light at 100 lux (LL) for 16 days. Actograms are double plotted on a 24 h time scale. Grey shading indicates periods of darkness. Activity data displayed in 1 min bins. (**B**) Comparison of period length (*tau*) in 12: 12 LD and LL after injection shows similar increase in period duration for *hOPN4 WT*, *Pro10Leu* and *Thr394Ile* treated mice. *** *P* < 0.001 Main effect of injection, two-way mixed ANOVA. (**C**) Period in LL is similar in all three *hOPN4* AAV conditions. *n* = 4–7 for each group. n.s. = not significant, one-way ANOVA. Data are shown ± S.E.M. Error bars smaller than symbols are not shown.

Another replicable phenotype of *Opn4*^*−*^^*/*^^*−*^ mice is attenuated period lengthening under constant light (LL) (2,4). We therefore used period lengthening in LL to assess levels of functional melanopsin restoration following injection of *hOPN4* SNP AAVs compared to *hOPN4 WT* controls. To avoid potential light-induced damage to the retina, which might have confounded the results after *hOPN4* AAV delivery, animals were only tested in constant light after injection following completion of the circadian screen (Fig. 4A). This prevented any comparisons of period in LL before and after injection. Nevertheless, the interaction of virus with period in LD and LL could still be assessed, with smaller increases in period length under constant conditions indicating disruption to melanopsin function, as could direct comparison of the period in LL of mice treated with *hOPN4* SNPs and *WT* control.

Period length (*tau*) in LL was significantly longer than in LD as anticipated, *F*(1, 13) = 50.79, *P* < 0.001 (Fig. 4B), with no significant main effect of virus, *F*(2, 13) = 0.13, *P* = 0.878, and more notably, no significant interaction of lighting condition with virus was observed on period, *F*(2, 13) = 0.21, *P* = 0.814, tested using a two-way mixed ANOVA with light condition as the within subjects variable (two levels—LL and 12: 12 LD cycle) and virus as the between subjects variable (three levels—*hOPN4 WT*, *Pro10Leu* and *Thr394Ile*). These data suggest the increase in *tau* seen in LL compared to LD was equivalent for all three virus conditions, and that the introduction of *hOPN4* SNPs did not affect melanopsin-associated period lengthening in LL. There was no difference in period under constant light between either *hOPN4 Pro10Leu* or *Thr394Ile*, and *hOPN4 WT* control, *F*(2, 15) = 0.17, *P* = 0.849 (one-way ANOVA, Fig. 4C), providing further evidence these SNPs do not disrupt melanopsin function sufficiently to negatively influence *hOPN4*-driven NIF behaviours.

### ipRGCs expressing *hOPN4 Thr394Ile* or *Pro10Leu* show abnormal melanopsin-driven responses to light compared to *hOPN4 WT* controls

**Figure 5.**
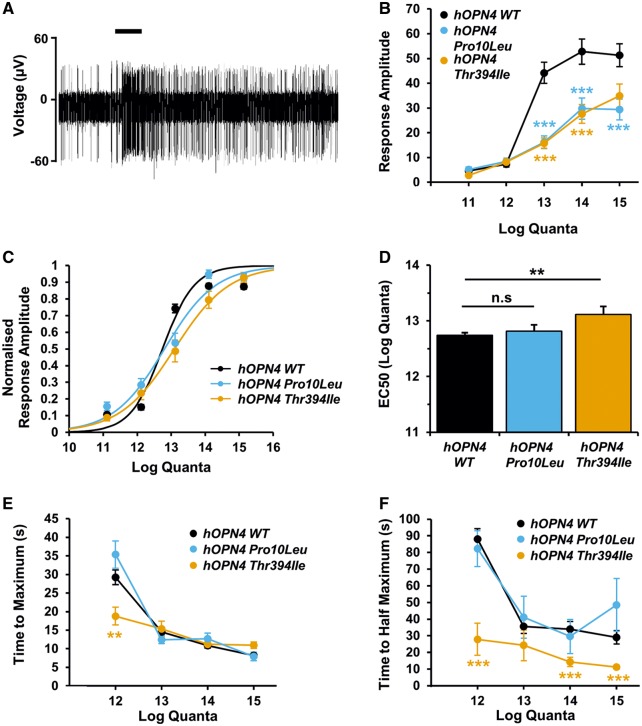
Abnormal response characteristics are shown by ipRGCs expressing *hOPN4 Thr394Ile* or *Pro10Leu* compared to *hOPN4 WT* AAV. (**A**) Example melanopsin-driven ipRGC responses to 10 s 480 nm light pulse (14 log quanta, shown as black bar above trace) during MEA recording of a *hOPN4 WT* AAV-treated *Opn4^−/−^ Cre^+/+^*retina during pharmacological blockade of rod/cone signals (**B**–**F**). ipRGC spike firing was recorded to increasing intensity 480 nm light (11.1–15.1 log quanta) (B) Response amplitude, defined as maximum increase in average firing rate over 10 s (spikes/s) above baseline. (C) Irradiance-response curves fit to normalized response amplitude to 480 nm 10 s light pulse (over 5-log range of intensities) using non-linear regression. (D) IpRGCs expressing *hOPN4 Thr394Ile* are less sensitive than *hOPN4 WT* control, shown by increase in mean EC50 value. No difference is observed between *hOPN4 WT* and *Pro10Leu*. (E) Time to maximum firing rate, a measure of response latency, is similar for all three *hOPN4* AAV conditions, except at low intensity light, whereas (F) time to half maximum firing rate, a measure of response offset, was significantly faster in *Thr394Ile* ipRGCs compared to *WT* control across a range of intensities, with no change observed in *Pro10Leu*-treated retina. Average ipRGC responses recorded from *Opn4^−/−^ Cre^+/+^* retina treated with *hOPN4 WT* (*n* = 83 from three retinas), *Pro10Leu* (*n* = 11 from two retinas) and *Thr394Ile* (*n* = 20 from two retinas) AAV *** *P* < 0.001 and ** *P* < 0.01 Sidak-corrected *t*-tests to explore significant virus by intensity interaction (two-way mixed ANOVA). Data are shown ± S.E.M. Error bars smaller than symbols are not shown.

To further examine whether *hOPN4 Pro10Leu* and *Thr394Ile* SNPs specifically affect melanopsin function, MEA recordings of AAV-treated *Opn4*^*−*^^*/*^^*−*^*Cre^+/+^* retina were conducted during pharmacological blockade of rod and cone inputs to isolate intrinsic melanopsin-driven ipRGC responses. Under these conditions, light-evoked responses were recorded from either *hOPN4 WT, Pro10Leu* or *Thr394Ile* AAV-treated retina, with relatively slow onset latency and prolonged post-stimulus firing (Fig. 5A), consistent with the properties of intrinsic ipRGC responses recorded from *Opn4^+/+^* retina (21,22), also see Supplementary Material, Figures S3 and S7.

We analysed ipRGC spike firing rates following stimulation with 10s 480 nm light of increasing intensities (11.1–15.0 log quanta). All three virus treatments resulted in intensity dependent effects of light on spike firing (Fig. 5B andSupplementary Material, Fig. S3). Changes in spike firing were rarely observed to the lowest light intensity tested (11.1 log quanta), but rapidly increased after light onset at higher intensities, *F*(1.5, 163.2) = 34.8, *P* < 0.001 (two-way Mixed ANOVA with Greenhouse Geisser Correction). ipRGCs expressing either *hOPN4 Pro10Leu* or *hOPN4 Thr394Ile* appeared to show smaller response amplitudes than *hOPN4 WT* at higher intensities. A significant virus by intensity interaction on response amplitude, *F*(3.0, 163.2) = 4.83, *P* = 0.003, was found to be driven by differences between *hOPN4 WT* and *Pro10Leu* at 13.1 (*P* < 0.001), 14.1 (*P* = 0.001) and 15.1 (*P* = 0.001) log quanta using post-hoc Student’s *t*-test (equal variance not assumed) with Sidak correction for multiple comparisons (new alpha = 0.0051). Significant differences were also found between *hOPN4 WT* and *Thr394Ile* at 13.1 (*P* < 0.001), 14.1 (*P* < 0.001). Differences for either SNP compared to WT at all other intensities were not significant (*P* > Sidak corrected alpha). Analysis of *Pro10Leu* is shown following removal of data from a single electrode that represented a statistical outlier (see Materials and Methods section for more details). Analysis of the complete *Pro10Leu* data and full description of the removed outlier can be found in Supplementary Material, Figures S4 and S5.

The overall sensitivity (measured as light intensity that produced half maximal response or EC50) of *hOPN4 WT*, *Thr394Ile* and *Pro10Leu* responses was calculated from a sigmoidal irradiance–response curve (IRC) fit to normalized maximum increase in firing rate across a 5-log range of intensities using non-linear regression (Fig. 5C). A one-way between subjects ANOVA revealed a significant difference in the EC50 values of the three virus conditions, *F*(2, 113) = 5.549, *P* = 0.005 (Fig. 5D), which a Dunnett’s *post-hoc* test confirmed was due to a significant decrease in sensitivity for *hOPN4 Thr394Ile* (EC50 = 13.1 log quanta) compared to *hOPN4 WT* (EC50 = 12.7 log quanta), *P* = 0.002. There was no difference in sensitivity between *hOPN4 Pro10Leu* (EC50 = 12.8) and *hOPN4 WT*, *P* = 0.857. Sensitivity was broadly consistent across different retina treated with each virus (Supplementary Material, Fig. S6). Overall, sensitivity of ipRGC light responses was moderately increased following *hOPN4* AAV treatment compared to responses recorded from native *Opn4^+/+^* retina under similar conditions (Supplementary Material, Fig. S7B and C).

Response onset and decay of ipRGC spike firing were quantified by examining time to peak firing rate and time to half-maximum firing rate, respectively (Fig. 5E and F). As shown in Figure 5E, all three virus conditions were slower to reach peak firing at lower light intensities, *F*(1.6, 164.2) = 46.5, *P* < 0.001. A significant virus by intensity interaction was also found, *F*(3.2, 164.2) = 4.39, *P* = 0.004 (two-way Mixed ANOVA with Greenhouse Geisser correction), which *post-hoc* Student’s *t*-test (equal variance not assumed) revealed was due to reduced onset latency in *hOPN4 Thr394Ile* compared to *hOPN4 WT* at 13.1 log quanta, *t*(48.0) = 3.34, *P* = 0.002 (Sidak correction for multiple comparisons, new alpha = 0.0064). Differences between *Thr394Ile* and *WT* at all other intensities were not significant (*P* > Sidak corrected alpha). No differences were found in the time to maximum firing rate of *hOPN4 Pro10Leu* and *hOPN4 WT* control (*P* > 0.05). As *hOPN4 Thr394Ile* did not differ from *hOPN4 WT* at other intensities, response onset appears broadly comparable between both *hOPN4* SNPs and *hOPN4 WT*.

At higher intensities, ipRGC responses demonstrate a rapid increase in firing rate followed by decay to a sustained period of slower spike firing (Supplementary Material, Fig. S3). Time to half-maximum firing rate was used as a measure of response decay and duration of melanopsin signalling. As with response onset, a significant main effect of intensity on response decay was found, *F*(2.3, 250.9) = 16.49, *P* < 0.001, driven by slower decay after low intensity light stimuli (Fig. 5F). A significant virus by intensity interaction, *F*(4.5, 250.9) = 3.51, *P* = 0.006, suggested this was driven by faster response decay in *hOPN4 Thr394Ile* that was not consistent across different intensities. *Post-hoc* Student’s *t*-tests (Sidak correction for multiple comparisons, new alpha = 0.0064) showed that, while *hOPN4 Pro10Leu* did not significantly differ from *hOPN4 WT* at any intensity (*P* > 0.05), *hOPN4 Thr394Ile* had significantly faster response decay than *hOPN4 WT* control at all intensities (*P* < 0.001) except 13.0 log quanta (*P* > 0.05). It is intriguing that *hOPN4 Thr394Ile* showed faster response decay to a wide range of light intensities but only relatively small changes to response onset, which were specific to low intensity light. This suggests there may be specific functional difference between *hOPN4 WT* and *Thr394Ile* affecting response offset kinetics.

### Levels of melanopsin expression are similar for *hOPN4 WT, Pro10Leu and Thr394Ile* AAV-treated retina

**Figure 6.**
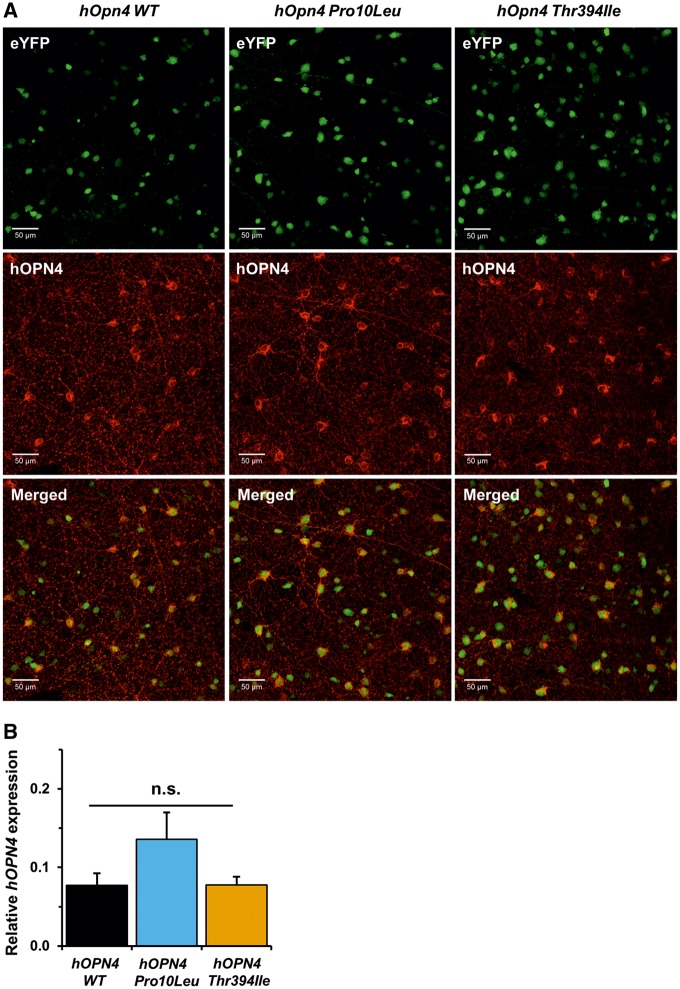
Expression of virally-delivered *hOPN4* is comparable between retina treated with *hOPN4 WT, Pro10Leu* and *Thr394Ile* AAV. (**A**) Transduction efficiency is comparable between the three *hOPN4* viruses, demonstrated by similar levels of co-localization of AAV-delivered hOPN4 (red) in ipRGCs labelled with genetic eYFP reporter (green) in injected *Opn4^−/−^ Cre^+/+^* retina. (**B**) Decreased sensitivity in ipRGCs expressing *hOPN4 Thr394Ile* is not due to differences in melanopsin expression level, which are comparable between the three *hOPN4* AAV conditions. Relative mRNA expression of *hOPN4* was normalized to four housekeeping genes (*Arbp, Gapdh, Psmb2* and *B2m*). *n* = 6–7 retina for each group. n.s. = non-significant, Main effect of virus, one-way ANOVA. Data are shown ± S.E.M.

RT-qPCR with *hOPN4* specific primers confirmed there were no differences in levels of *hOPN4* mRNA expression in the retina of *Opn4*^*−*^^*/*^^*−*^*Cre^+/+^*animals 18–20 weeks after injection with *hOPN4 WT*, *Pro10Leu* or *Thr394Ile* AAV, *F*(2, 18) = 1.667, *P* = 0.220 (one-way ANOVA, Fig. 6B). Immunohistochemistry of AAV-treated retinas also showed similar levels and distribution of anti-hOPN4 staining between the three virus groups (Fig. 6A). No differences in cellular distribution were observed with hOPN4 *WT, Pro10Leu* and *Thr394Ile* proteins all successfully trafficked to the cell membrane and along cellular processes. These data suggest that transduction efficiency was similar between the different viruses, and that the attenuated sensitivity observed in *Opn4*^*−*^^*/*^^*−*^*Cre^+/+^*mice injected with *hOPN4 Thr394Ile* AAV was not due to differences in overall levels of *hOPN4* mRNA or protein expression.

## Discussion

We have used targeted delivery of WT or mutant *hOPN4* AAV to mouse ipRGCs *in vivo* to model the effects of *hOPN4* SNPs, *Pro10Leu* and *Thr394Ile*, on a range of NIF responses to light. The *hOPN4-*AAV animal model allows, for the first time, the functional effects of *hOPN4* SNPs to be investigated in detail, building on previous studies in humans which have identified genetic associations between the occurrence of these polymorphisms and potential NIF phenotypes (7,9–13). This novel implementation of an *in vivo* functional rescue approach was first validated, with ipRGC-specific delivery of *hOPN4* shown to functionally rescue ipRGC light responses and known melanopsin-associated behaviours, such as pupil constriction and phase shifting to nocturnal light pulses (2–4,19,20).

This functional rescue approach results in an overall increase in melanopsin expression throughout the virally treated retinas. However, levels of melanopsin expressed within M1 type ipRGCs, responsible for circadian entrainment and PLR (23), are likely much closer to physiological levels. Under native conditions, different ipRGC subtypes exhibit heterogeneity in levels of melanopsin expression. M1 cells represent just 20% of all ipRGCs (15), but express significantly higher levels of melanopsin (10–40-fold) than M2 type ipRGCs (24,25), with levels even lower in M4 and M5 cells and typically below the threshold for detection (15,16). This characteristic pattern of melanopsin expression is not replicated following AAV delivery, where both M1 and non-M1 cells express similar levels of melanopsin. The increased expression in non-M1 cells (80% of all ipRGCs) likely accounts for much of the increase in total retinal melanopsin, and the overall increase in sensitivity of ipRGC light responses observed in injected retinas compared to native conditions. Based on the sensitivity of individual MEA electrodes, our data indicate that all ipRGC subtypes in AAV-treated retina express melanopsin at levels similar to that observed in native M1 type ipRGCs and that the sensitivity of predominantly M1-driven behaviours, such as PLR, is not significantly enhanced in virus-treated animals compared to untreated WT animals.

Using the *hOPN4-*AAV animal model, no significant changes in PLR or circadian photoentrainment were identified in animals expressing either *hOPN4 Thr394Ile* or *Pro10Leu*, which were equally effective as *hOPN4 WT* at rescuing multiple melanopsin-dependant NIF behaviours. However, analysis of melanopsin-driven ipRGC light responses recorded using MEAs in the presence of pharmacological blockade of synaptic input from rods and cones revealed differences in sensitivity, response amplitude and offset kinetics of *hOPN4 Thr394Ile*-expressing ipRGCs compared to *hOPN4 WT* controls. By comparison, the sensitivity and kinetics of ipRGC light responses were unaffected by introduction of the *Pro10Leu* polymorphism, although response amplitudes of ipRGCs expressing this SNP were significantly attenuated.

Overall our data indicate that *hOPN4* SNPs *Pro10Leu* and particularly *Thr394Ile* have a direct effect on melanopsin function. The impact of *Thr394Ile* was only observed following analysis of melanopsin-driven ipRGC light responses, but did not result in detectable differences in NIF behaviour. The NIF behaviours chosen for testing *hOPN4* SNPs are known to be attenuated in *Opn4*^*−*^^*/*^^*−*^ mice, but these behavioural phenotypes can be relatively subtle due to high levels of functional redundancy caused by the overlapping roles of rods, cones and melanopsin in driving ipRGC light responses and inputs to NIF pathways (5,6). This synaptic input to ipRGCs from rods and cones may effectively limit the impact that melanopsin loss of function can have on behaviour. By contrast, MEA measurements of ipRGC light responses, performed during pharmacological blockade of confounding rod and cone inputs, can fully isolate melanopsin-dependent responses. The cellular electrophysiology approach therefore provides a direct assay of intrinsic melanopsin function, which allows more subtle changes in melanopsin activity to be detected compared to behavioural assays.

In the case of *hOPN4 Thr394Ile*, we found considerable evidence of abnormal melanopsin activity, with ipRGC responses showing attenuated sensitivity and response amplitude, as well as faster response decay, compared to *hOPN4 WT* controls. These data suggest *Thr394Ile* is a functionally significant site for normal melanopsin signalling. In support of this conclusion, *Thr394Ile* is located within a conserved cluster of serines and threonines located in the C-terminus, recently implicated as phosphorylation sites involved in the deactivation kinetics of melanopsin responses (26–28). Alternatively, it has been suggested that interactions between proximal and distal regions of the C-terminus are responsible for maintaining the equilibrium between active and inactive states necessary for the sustained signalling and temporal integration exhibited by melanopsin (27,29). Therefore, it is possible that *Thr394Ile* could affect protein stability as it transitions between active and inactive states, shifting the balance toward inactive states and disrupting normal melanopsin activity.

In contrast to *Thr394Ile*, we found more limited evidence to indicate that the *Pro10Leu* SNP directly influences melanopsin function at the behavioural or ipRGC level. Delivery of *hOPN4 Pro10Leu* AAV led to a complete functional rescue of melanopsin-dependent NIF behaviours in *Opn4*^*−*^^*/*^^*−*^*Cre^+/+^*mice, comparable to *hOPN4 WT* AAV. Furthermore, the sensitivity, and response kinetics of MEA-based responses were comparable between ipRGCs expressing *hOPN4 Pro10Leu* and *hOPN4 WT*. However, we did observe reduced response amplitude in cells expressing *Pro10Leu* compared to *hOPN4 WT*. Overall, these data suggest *Pro10Leu* has a minor impact on melanopsin function. This partially supports the conclusions of genetic association studies implying *Pro10Leu* is a genetic risk factor for SAD, altering melanopsin function and reducing photoreception. The lack of any behavioural phenotype in our data could reflect the fact that standard assays of NIF responses to light in mice fail to replicate complex human behaviours, such as sleep-wake timing or SAD, and indeed animal models of SAD are currently lacking. Given the genetic heterogeneity and difficulty controlling confounds in studies of complex multi-factorial disease, it is also possible the association of *Pro10Leu* with SAD is due to genetic risk factors for SAD that are in linkage disequilibrium with *Pro10Leu*. Segregation of *Pro10Leu* with other such risk factors could lead *Pro10Leu* to occur with a higher frequency in individuals with the SAD phenotype, with only a minor contribution to the condition.

This study raises interesting questions about how polymorphisms in *hOpn4* may underlie phenotypes related to NIF responses to light. Unlike rod and cone opsins, missense mutations in melanopsin are unlikely to give rise to deleterious consequences, such as retinal degeneration and congenital stationary night blindness, which are associated with missense variants in rhodopsin (30–32). First, melanopsin is expressed at relatively low levels and does not play a structural role within ipRGC photoreceptors, making loss of function or misfolding melanopsin mutants unlikely to cause degeneration of ipRGCs. Second, in the absence of melanopsin phototransduction, light input to NIF regions of the brain is sustained by synaptic input to ipRGCs from rods and cones, and thus loss of function *hOPN4* SNPs may only result in minor disruption of overall ipRGC light responses and NIF behaviours.

In summary, we demonstrate that naturally occurring polymorphisms in *hOPN4* can be studied *in vivo* to determine the consequences of specific mutations on melanopsin function and associated NIF behaviours. We show that *hOPN4* SNPs *Thr394Ile* and *Pro10Leu* are directly associated with altered melanopsin signalling, although these effects are not sufficient to exert significant influence on NIF behaviours in mice with healthy retinas containing functional rods and cones. To our knowledge, this represents the first direct evidence for human genetic polymorphisms giving rise to altered melanopsin function and provides further insight into the expected effects of *hOPN4* SNPs on human physiology and NIF behaviours. This approach offers significant advantages over previous methods used to study melanopsin function, such as heterologous expression using *in vitro* cell line systems, and will be invaluable for determining the functional consequences of other *hOPN4* SNPs and functional mutants.

## Materials and Methods

### Amino acid alignment

Melanopsin protein sequences from the following species were used for multiple alignment—*Homo sapiens* (NP_150598.1, NP_001025186.1), *Pan troglodytes* (XP_001135445.1, XP_001135533.1), *Macaca mullata* (XP_001088248.2), *Canis familiaris* (XP_853735.2), *Bos taurus* (NP_001179328.1), *Felis catus* (AAR36861), *Mus musculus* (NP_038915.1, NP_001122071.1), *Rattus Norvegicus* (NP_620215.1), *Phosopus sungorus* (AAU11506), *Nannospalax ehrenbergi* (CAO02487), *Gallus gallus* (ABX10832.1, ABX10833.1, ABX10834.1, NP_989956.1, ABX10831.1), *Sminthopsis crassicaudata* (ABD38715), *Danio rerio* (NP_001245153.1, ADN39430, NP_840074.1, NP_001243006.1, ADN39434.1), *Podarcis siculus* (AAY34941.2), *Xenopus laevis* (NP_001079143.1) and *Brachiostoma belcheri* (BAE00065). Multiple sequences for a given species represent different splice variants or distinct melanopsin genes, which are duplicated in non-mammals (33). Protein sequences were aligned using multiple sequence alignment software, MAFFT (34).

### Animals

All experiments were approved by the University of Oxford Animal Welfare and Ethical Review Board and all procedures were conducted in accordance with the UK Home Office Animals (Scientific Procedures) Act 1986 (Project Licences 30/2812 and 30/3371, PILs I72E6A362 and IB2F9F14B). Six *Opn4^+/+^ tau-LacZ*^*−*^^*/*^^*−*^ and 6 *Opn4*^*−*^^*/*^^*−*^*tau-LacZ*^+/+^ mice (1) were used as controls and 59 *Opn4*^*−*^^*/*^^*−*^*Cre^+/+^ eYFP*^+/+^ mice were used for AAV experiments. Both lines were maintained on a mixed C57BL/6 and 129/SvJ background. The *Opn4.Cre.eYFP* line was generated as described previously (15) by breeding *Opn4.Cre* mice (16) with mice containing a lox.STOP.lox eYFP sequence inserted within the Rosa26 locus (35), resulting in expression of Cre recombinase and eYFP within M1-M5 subtypes of ipRGCs (15). As Cre-recombinase replaces the *Opn4* open reading frame in these mice, *Opn4*^*−*^^*/*^^*−*^*Cre^+/+^ eYFP*^+/+^ animals lack endogenous melanopsin expression. Unless stated otherwise, all mice were kept under a 12: 12 light dark cycle with food and water provided *ad libitum*. All experiments were conducted double-blind, with animals randomly allocated to different virus treatment groups before injection. Injections, data collection and analysis were then completed before virus groups were unmasked.

### Virus production

Three viruses were produced: AAV*2-DIO-hOPN4 WT*, AAV*2-DIO-hOPN4 Pro10Leu* and AAV*2-DIO-hOPN4 Thr394Ile*. An AAV2/2 capsid with four tyrosine to phenylalanine mutations (36) was used to achieve efficient viral transduction of retinal cells, in particular in retinal ganglion cells (37). The *hOPN4* AAV plasmid was constructed by replacing the inverted ChR2-mCherry sequence in pEF1α-AAV-DIO-ChR2-mCherry (38) with an inverted hOPN4 sequence (NM_033282.3) fused to a 1D4 tag, using restriction enzyme cloning. Single base point mutations were introduced by site directed mutagenesis with QuikChange II XL kit (Stratagene) using the following primers; *Pro10Leu* F 5′–CCAAGAGTCCTGCCCAGCCCAACCC, *Pro10Leu* R 5′–TTGG GCTGGGCAGGACTCTTGGC, *Thr394Ile* F 5′-CAGCATCCCCATGAGTCCCACCAGCAAGAT and *Thr394Ile* R 5′- ATCTTGCTGGTGGGACTCATGGGGATGCTG. The inverted *hOPN4* transgene is flanked by two pairs of Lox sites (LoxP and Lox2272) so that in the presence of Cre recombinase, the transgene is inverted into the sense orientation and expression is driven by a constitutive EF1α (elongation factor 1α) promotor (39).

Viruses were produced using method described previously (37). Hek293T cells were transfected with three plasmids: *pEF1a-DIO-hOPN4–1D4-WPRE* (containing inverted terminal repeats, woodchuck hepatitis B virus post-transcriptional regulatory element, and transgene), *p*AAV*2*/2 (*quad Y-F*) (containing *rep* and *cap* genes) and *pAdΔF6* (containing helper genes from adenovirus genome) and harvested 72 h later. Cell pellets were lysed in 0.15 m NaCl, 50 mm Tris–HCl (pH8.5) and 500µl EDTA-free protease-inhibitor cocktail (Roche) in PBS, then underwent freeze-thaw cycles ×4 at −80°C and 37°C, before incubation with benzonase (50 U/ml, Sigma-Aldrich, St. Louis, MO, USA) for 30 min at 37°C to remove cell DNA contamination. AAV particles were then purified by iodixanol gradient ultracentrifugation and concentrated using Amicon Ultra-15 centrifuge filters (Merck-Millipore, Burlington, MA, USA). SDS-PAGE was used to confirm virus purity, and the titre of DNase1-treated virus was determined by standard-curve qPCR using primers designed to amplify *hOPN4* (forward 5′–GTTGCTGACATCCTGCTCCT, reverse = 5′-TCCCGGATGGCCCTGAAGAT).

Where appropriate, the titre of each virus was adjusted by dilution in PBS to 2.2 × 10^12^ viral genomes/ml. and aliquots stored at −80°C prior to use. For experiments examining the impact of viral titre, a medium (2.2 × 10^11^ viral genomes/ml) and low (2.2 × 10^10^ viral genomes/ml) titre were also prepared.

### Intravitreal injections

Animals were anaesthetized by intraperitoneal injection of xylazine (10 mg/kg body weight) and ketamine (80 mg/kg body weight). Pupils were dilated with 1% tropicamide and 2.5% phenylephrine hydrochloride eye drops (Bausch & Lomb, Rochester, NY, USA) and a 6 mm coverslip positioned on gel lubricant (Viscotears, Novartis, Basel, Switzerland) was applied to the cornea. 1.5 µl of virus at 2.2 × 10^12^ virus particles/ml was injected into the vitreous using a Hamilton syringe with a 10 mm 34-gauge needle (65N, Hamilton AG, Bonaduz, Switzerland) using a surgical microscope (M620 F20, Leica, Wetzlar, Germany). All animals received bilateral intravitreal injections of the same virus. Anaesthesia was reversed by intraperitoneal injection of atipamezole (10 mg/kg body weight). During recovery, 0.5% proxymetacaine hydrochloride and 0.5% chloramphenicol (Bausch & Lomb) was applied to injected eyes. All animals began testing 1 month after virus injection.

### Pupillometry

Pupillometry was performed as described previously (40). Animals were housed on a 12: 12 light: dark cycle and dark adapted for a minimum of 1 h prior to testing at ZT 4–8. During pupillometry, mice were manually restrained by scruffing (not anaesthetized). A 1% tropicamide solution (Bausch & Lomb) was applied to the light exposed eye 10–15 min before first recording. The consensual pupil response was recorded from the contralateral eye, which was not directly exposed to light. Light stimuli were generated using a xenon arc lamp (150 W solar simulator, Lot Oriel, Darmstadt, Germany) filtered using monochromatic bandpass filters (480 or 600 nm, both 10 nm half-bandwidth, Andover, Salem, NH, USA), and transmitted to the stimulated eye via a liquid light pipe as an irradiant light stimulus using a 5 cm Ganzfeld integrating sphere (Pro-lite Technology, Cranfield, UK). Light exposure was regulated by a shutter (LSZ160, Lot Oriel) positioned in the light path and controlled by custom software (BRSL, Newbury, UK). For recording of pupil images, the contralateral eye was illuminated by an infrared light emitting diode (850 nm, 10 nm half bandwidth) and images collected at 10 frames/s using a near infra-red sensitive CCD camera (Prosilica, BRSL).

All recordings consisted of 2 s of dark, 10 s of 480 nm (14.68 log quanta) or 600 nm (14.7 log quanta), then up to 40 s of dark, to examine dark-adapted pupil size, pupil constriction and recovery from constriction, respectively. Any animals which did not show pupil constriction were excluded from further testing and data analysis (2 of 59 mice). Images were analysed using ImageJ (http://rsbweb.nih.gov/ij; date last accessed May 2018). Minimum pupil constriction was measured by manually fitting a circle to the pupil for the first frame of recording and the last frame of light exposure, then calculating the area of both circles. The area of the pupil at the end of light stimulus was then normalized to dark-adapted pupil area to give a maximum constriction value. For pupillometry comparing mice injected with *hOPN4 WT* and *hOPN4* SNP AAVs, four technical replicates were conducted for each animal. These technical replicates were all analysed as described above and then averaged to create a single minimum pupil area value for each animal, which was compared between groups. Kinetic traces of pupil area shown in Figure 2A and Supplementary Material, Figure S2A represent averages of representative traces for each animal. Rate of pupil recovery was assessed by fitting a linear trendline to representative normalized pupil size data for each animal from 0–15 s after light offset. In cases where pupil recovery data were missing due to animal movement, the trendline was fitted to available data. The slope of best-fit trendline was then used as a measure of rate of recovery.

### Passive-infrared activity monitoring

PIR activity monitoring was conducted as described previously (41). Animals were housed individually in large cages (44 cm × 26 cm × 12 cm) with a transparent acrylic block placed under the food hopper to keep animals within PIR detection range at all times. Six cages were arranged in each light-tight chamber with light provided by white LEDs of variable intensity. A pyroelectric (passive) infra-red detector (PIR) was positioned above the centre of each cage to detect motion. Raw data was collected from each PIR every 100 ms for 10 s and then averaged to give a percentage active value every 10 s. Raw PIR data was then resampled into 1 min bins using custom Python scripts and circadian parameters analysed with Clocklab (Actimetrics, Wilmette, IL, USA) for Matlab (Mathworks, Natick, MA, USA). Phase shifting experiments were conducted before and 1 month after injection of *hOPN4* AAV. Activity was first recorded under a 12: 12 light: dark cycle (LD) under 350–400 lux light (14.5–14.6 log quanta) light for at least 7 days, then in constant darkness (DD) for a minimum of 11 days. On the first day of DD a 15 min 100 lux (13.94 log quanta) white LED light pulse was given at ZT15 to induce a phase shift. Activity under constant light (LL) was only recorded after injection, to avoid confounding effects of injection with possible damage to retina from LL. Animals were placed in 12: 12 LD for 7 days before being released into 100 lux constant white light (LL) for 14 days to monitor period lengthening.

### Multi-electrode array recordings

MEA recordings were performed as described previously (42). Animals were dark adapted for 1 h, culled and enucleated, and retinas dissected under dim red light in Ames media (pH 7.4, Sigma) gassed with 95% O_2_ 5% CO_2_, and positioned ganglion cell-side down onto glass-based MEA chambers with 60 electrodes (30 μm Ø, 200 μm apart, Multi Channel Systems, Reutlingen, Germany) and secured by glass-coated metal harps (ALA Scientific Instruments). MEA chambers were then mounted into a MEA amplifier (MEA2100–2 × 60 system, Multi Channel Systems) positioned onto the stage of an Olympus IX71 inverted microscope with recording electrodes positioned in the microscope light path. MEA chambers were then attached to a gas-permeable perfusion manifold (ALA Scientific Instruments, Farmingdale, NY, USA) and perfused continuously with AMES media gassed with 95% O_2_ 5% CO_2_ (pH 7.4) heated to 34°C for 15–30 min, before being switched to media containing 100 μm l(+)-2-amino-4-phosphonobutyrate (L-AP4) (group III metabotropic glutamate receptor agonist), 40 μm 6, 7-dinitroquinoxaline-2, 3-dione (DNQX) (AMPA/kainate receptor antagonist), and 30 μm d-2-amino-5-phosphonovalerate (d-AP5) (NMDA receptor antagonist) (all from Tocris, Bristol, UK), to block synaptic input from rod and cone photoreceptors and isolate endogenous ipRGC light responses (43). Following a further 30 min of dark adaption, retina were first stimulated with a test pulse of 480 nm ±10 nm (14.1 log quanta) light for 10 s, then dark adapted for 15 min to produce an equal dark adaptation baseline for subsequent experiments. Irradiance response curves (IRCs) were performed by exposure to increasing intensities of 10 s 480 nm light ranging from 11.1 to 15.1 log quanta. Activity was recorded for 30 s prior to light stimulation (baseline), then for 2 min after onset of light exposure. Periods of dark adaptation between pulses ranged from 2 to 15 min depending on the intensity of preceding light stimulation (38).

Light stimuli were generated by an X-cite 120 W metal halide light source (EXFO) and 480 nm ±10 nm band-pass filter (ThorLabs, Newton, NJ, USA) delivered via a ×10 microscope objective beneath the MEA chamber. Duration of light stimuli was manually controlled by a high-speed shutter (Prior Scientific, Cambridge, UK). Intensity of light stimuli was adjusted using neutral density filters (0–5 log units, ThorLabs) controlled via an automated filter wheel (Prior Scientific). Power of light stimuli (µW/cm^2^/s) were measured at the sample focal plane using an in-line power meter (PM160T, ThorLabs) and converted to photon flux (log quanta) using an irradiance conversion toolbox (see supplementary material in 6). Electrode signals were collected, amplified and digitized at 25 kHz using MC Rack software (Multi Channel Systems). Analysis of spike firing rates was performed offline using MC rack software (Multi Channel Systems). Raw electrode data was filtered using a 200 Hz high-pass filter and spikes detected using an amplitude threshold of 3 standard deviations from baseline. Spikes were then sorted into 1 s bins (spikes/s) and analysed using custom Excel templates. Electrodes with ipRGC activity were identified by an increase in maximum average firing rate over 10 s that was greater than 10 spikes/s after exposure to a 14.1 log quanta 480 nm light.

The following response parameters were calculated for each recording: Response amplitude was defined as maximum increase in firing rate (spikes/s) averaged over 10 s from baseline firing rate. Baseline firing rate was defined as average firing rate in 10 s before light onset. Given the relatively slow kinetics of melanopsin-driven activity, maximum 10 s average firing rate was used to provide a reliable measure of intrinsic ipRGC spike firing and prevent response amplitude from being overly influenced by short bursts of high frequency firing that may be the result of residual rod or cone activity or fluctuations in spontaneous background firing rate. For IRCs, a two-parameter sigmoid function: *y* = 1/(1 + 10^*a*^^(^^*b*^^−^^*x*^^)^), where *a* = Slope and *b* = EC50 (Log Quanta) was fit to response amplitude normalized to maximum for each ipRGC. The best-fit EC50 values from each IRC were then averaged and compared between groups as a measure of sensitivity. IRCs were fit to data using non-linear regression. Time to maximum firing rate, a measure of response onset, was defined as time (s) from light onset to maximum firing rate. Time to half maximum firing rate, a measure of response offset, was defined as time (s) from peak firing rate to half-maximum firing rate. During analysis of response amplitude for individual ipRGCs expressing *hOPN4 Pro10Leu*, we identified an outlier with response amplitude at 15.1 and 14.1 log quanta that was ∼3 standard deviations larger than the mean. Data from this cell were excluded from further analysis of *Pro10Leu* response characteristics. Full details of this outlier and analysis of the response characteristics of *Pro10Leu*-expressing ipRGCs including this data are shown in Supplementary Material, Figures S4 and S5. No equivalent statistical outliers were identified for cells expressing *hOPN4 WT* or *Thr394Ile.*

### Immunohistochemistry

Immunohistochemistry was performed as described previously (15,40). Whole eyes were pierced with a 28-gauge needle, fixed in 4% formaldehyde (Thermo Fisher, Waltham, MA, USA) in PBS at 4°C for up to 24 h, then transferred to 30% (w/v) sucrose in PBS and stored at 4°C for >48 h. Retinas were dissected from cryoprotected eyes and permeabilized in PBS with 1% Triton-X in PBS for 1 h at room temperature. Retinas were blocked in PBS with 10% donkey serum (Sigma-Aldrich) in PBS with 1% Triton X for 1 h, then incubated in chicken polyclonal anti-GFP, which also recognizes eYFP (GFP-1020, AVES Labs, Tigard, OR, USA, 1: 1000) and rabbit polyclonal anti- hOPN4 (sc-32870, Santa Cruz, Dallas, TX, USA, 1: 250) primary antibodies diluted in PBS with 2.5% donkey serum and 1% Triton-X for 72 h at 4°C. Secondary antibodies were donkey anti-chicken Alexa-488 (Jackson ImmunoResearch, West Grove, PA, USA) and donkey anti-rabbit Alexa-568 (Thermo Fisher) diluted 1: 200 in PBS with 2.5% donkey serum and 1% Triton-X for 16 hours at 4°C. All wash steps were performed in PBS with 1% Triton-X. Retinas were counterstained with DAPI (0.5 µg/ml) for 10 min at room temperature and mounted in Prolong Gold anti-fade reagent (Thermo Fisher). Fluorescent images were acquired using an inverted LSM 710 laser scanning confocal microscope (Zeiss, Oberkochen, Germany) with Zen 2010 image acquisition software (Zeiss) with excitation at 405, 488 and 561 nm and emission at 440–480, 505–550 and 600–700 nm for DAPI, green and red fluorescence respectively. Images were collected every 1 µm in the z-plane and merged to form full-depth images. Global enhancements of brightness and contrast were performed using ImageJ software. All images presented for comparison were processed under identical conditions.

### RT-qPCR

Animals were culled, enucleated and their retina immediately dissected and stored in RNAlater (Thermo Fisher). RNA was extracted using RNeasy mini kit (Qiagen, Hilden, Germany) and cDNA generated using qScript cDNA synthesis kit (Quanta Biosciences, Beverly, MA, USA), according to the manufacturer’s instructions. RT-qPCR was performed using Quantifast SYBR green reagents (Qiagen) using an Step-One Plus real-time PCR system (Applied Biosystems, Foster City, CA, USA) with Step-One software (version 2.1)**.** For all reactions cycling conditions were: 10 min at 95°C, followed by 40 cycles of 15 s at 95°C, 1 min at 60°C and 15 s at 76°C, then 15 min at 95°C and 60 s at 60°C. For comparison of *hOPN4* with endogenous mouse *Opn4 (mOpn4)* expression primers were designed to amplify both human and mouse melanopsin, F = 5′-TATGCCCTGGCCTGGAGTCT, R = 5′-TAGTCCCAGGAGCAGGATGT. For comparison of expression between different *hOPN4* AAVs, human *OPN4* specific primers were used, F = 5′-GTTGCTGACATCCTGCTCCT, R = 5′-TCCCGGATGGCCCTGAAGAT. Levels of *Opn4* expression are shown normalized to the geometric mean of multiple housekeeping genes: *Arbp* (Acidic ribosomal phosphoprotein P0), *Gapdh* (Glyceraldehyde-3-Phosphate Dehydrogenase), *β2m* (Beta-2-Microglobulin) and *Psmb2* (proteasome subunit—beta type 2) using method described in Vandesompele *et al.* (44). Primer sequences for housekeeping genes can be found in Jagannath *et al.* (45).

### Statistical Analysis

Statistical tests were conducted using SPSS 22.0 (IBM, Armonk, NY, USA). Unless stated otherwise, all comparisons between genotypes or virus treatments were tested using One-way between-subjects analysis of variance (ANOVA) with virus or genotype as the independent variable. All comparisons before and after injection for the three virus groups were tested using two-way mixed ANOVA with virus type as a between-subjects factor (three levels—*hOPN4 WT, Pro10Leu and Thr394Ile*) and injection as a within-subjects factor (two levels—before and after injection). Subsequent post-hoc tests to explore significant main effects or interactions were conducted on case-by-case basis and are detailed in the results section. Unless stated otherwise, a significance threshold of *P* ≤ 0.05 was used. In all figures * indicates *P* < 0.05, ** *P* < 0.01 and *** *P* < 0.001. All data are shown as mean ± standard error of the mean (S.E.M.). In all figures error bars smaller than symbols are not shown.

## Supplementary Material

Supplementary Material is available at *HMG* online.

## Supplementary Material

Supplementary Data
