# Supplementary figures and images for: Defining the impact of melanopsin missense polymorphisms using in vivo functional rescue
Source: Hum Mol Genet. 2018 Apr 30;27(15):2589–603. doi: 10.1093/hmg/ddy150 (PMC6048994; doi:10.1093/hmg/ddy150)

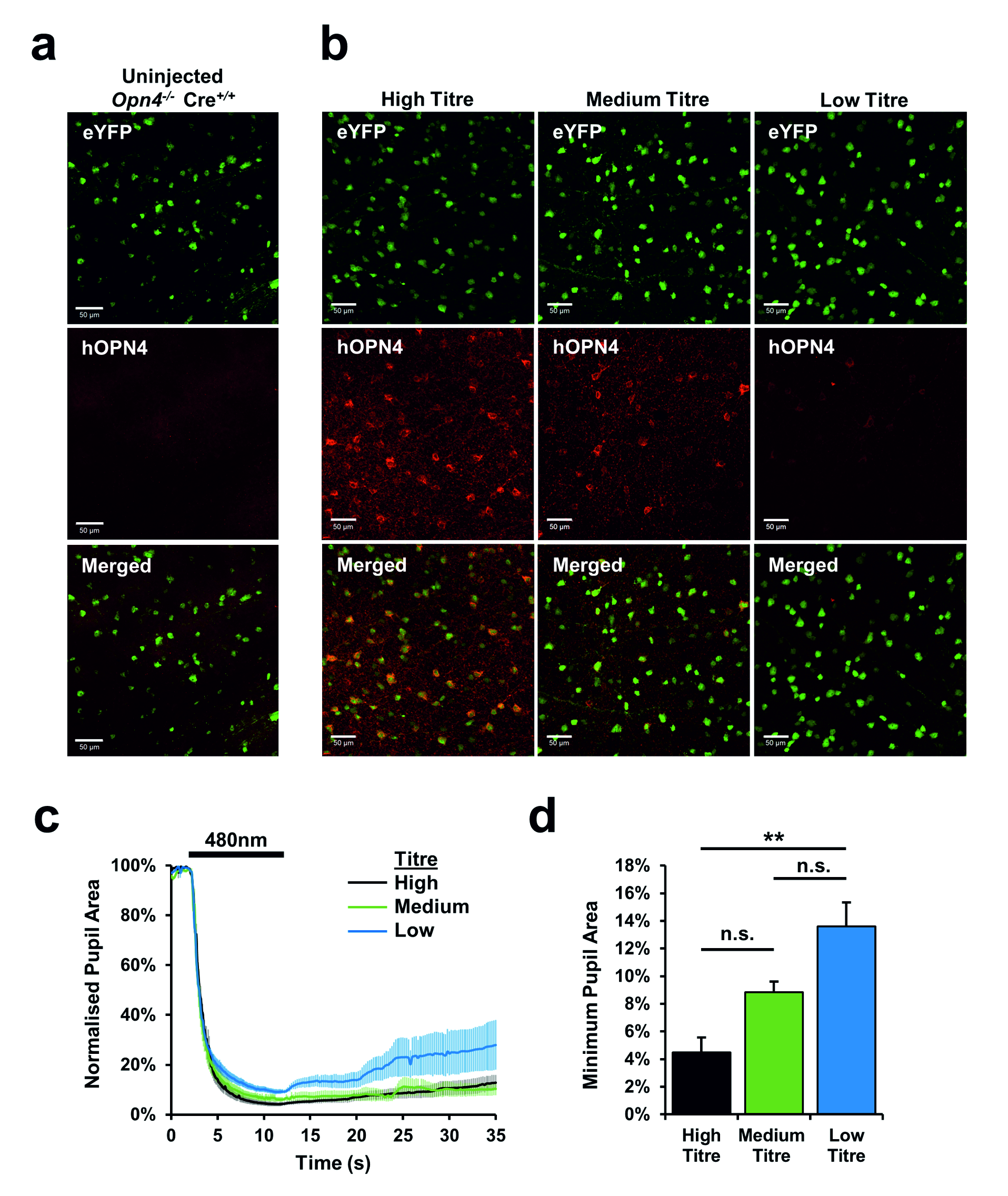

Supplement: Supplementary Data [file ddy150_supp.zip › ddy150-suppl_data/FigS1.tif]

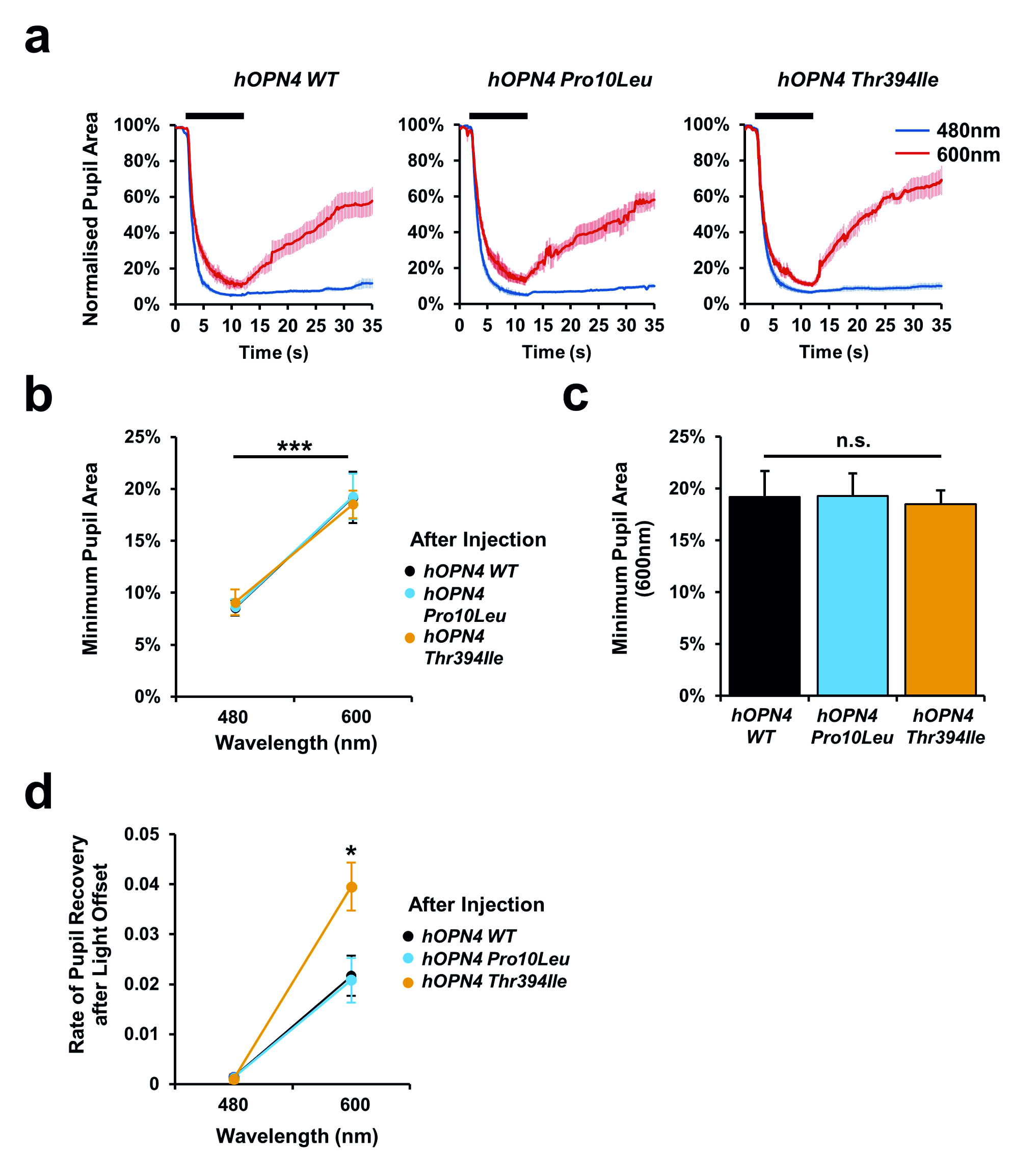

Supplement: Supplementary Data [file ddy150_supp.zip › ddy150-suppl_data/FigS2.tif]

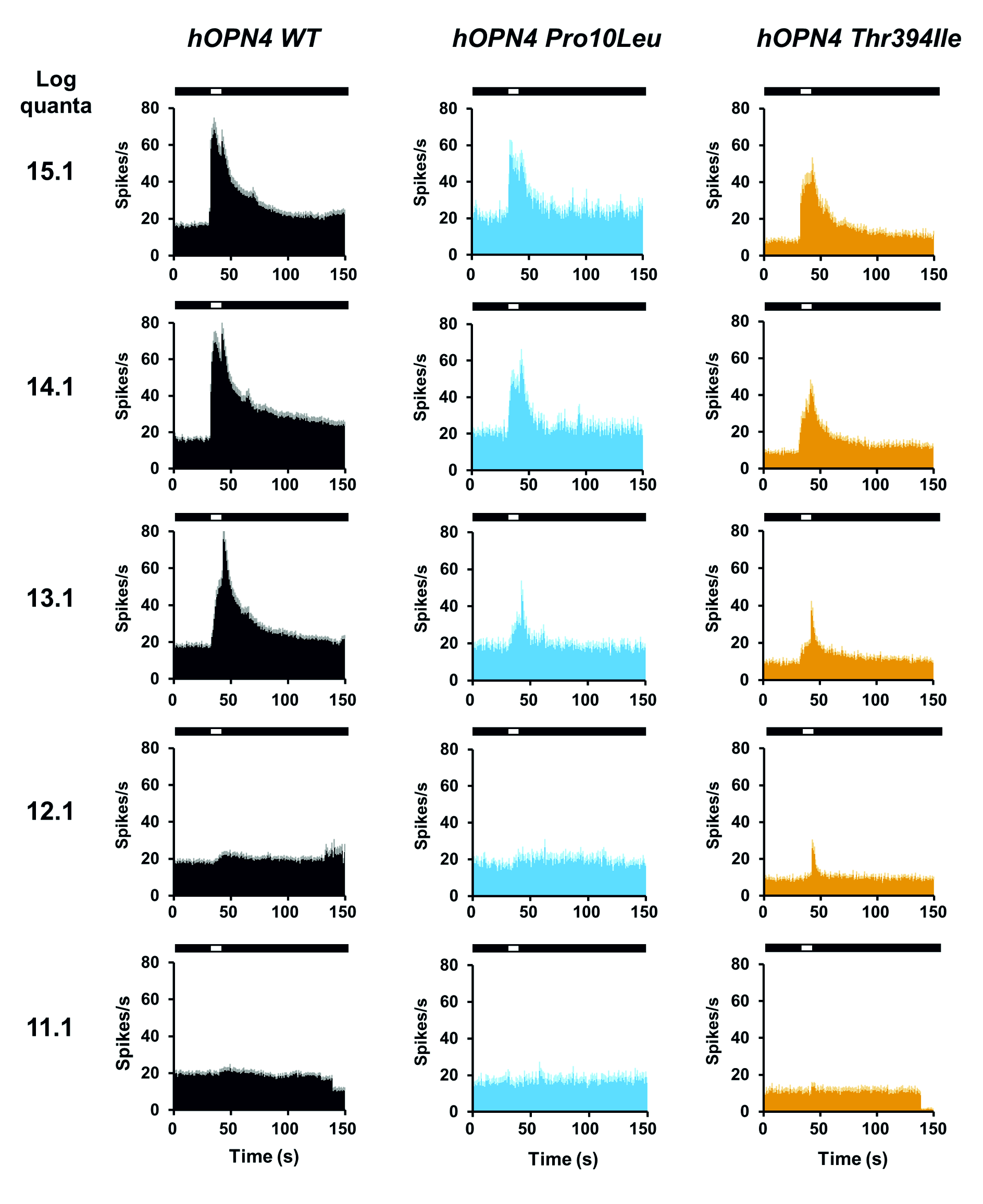

Supplement: Supplementary Data [file ddy150_supp.zip › ddy150-suppl_data/FigS3.tif]

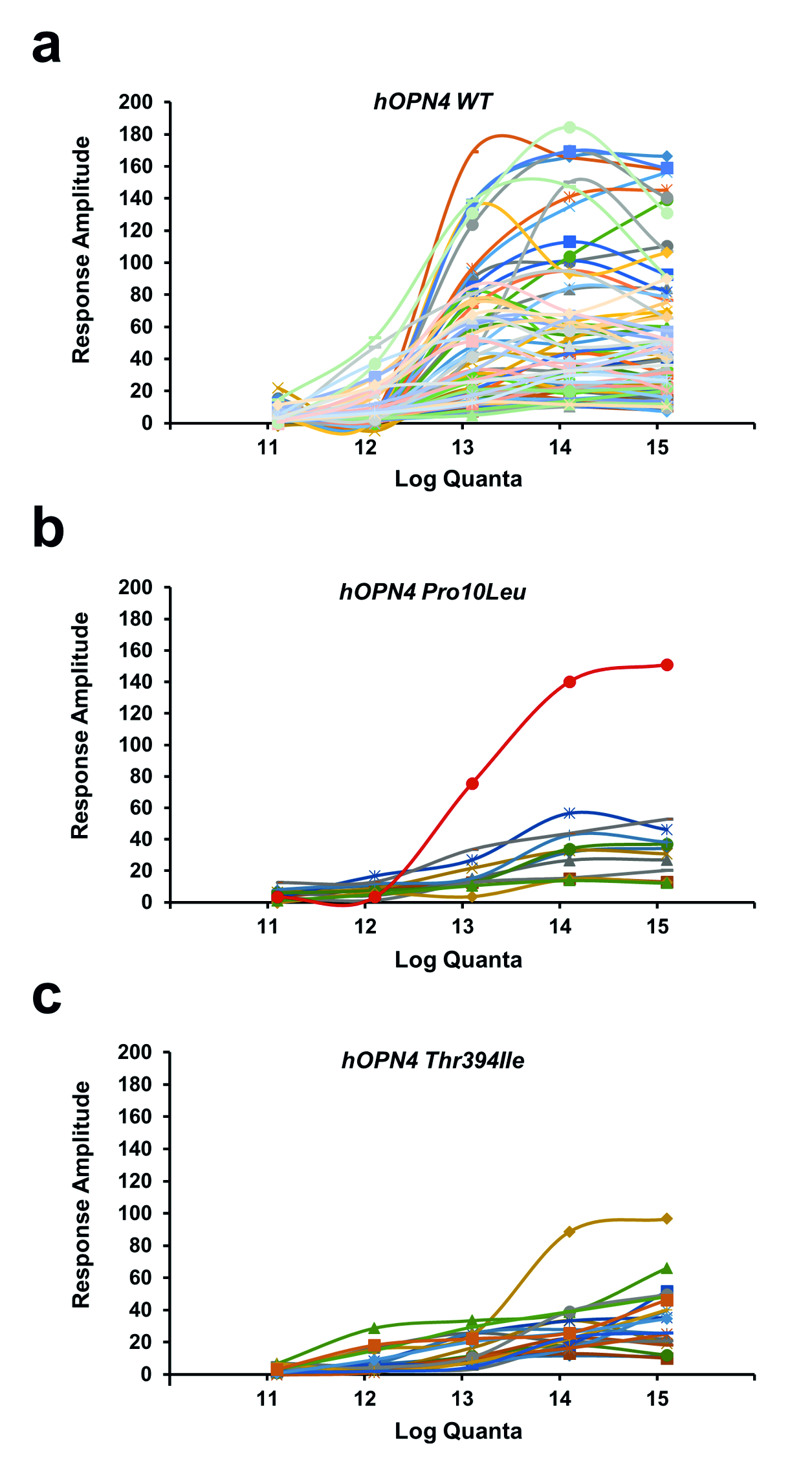

Supplement: Supplementary Data [file ddy150_supp.zip › ddy150-suppl_data/FigS4.tif]

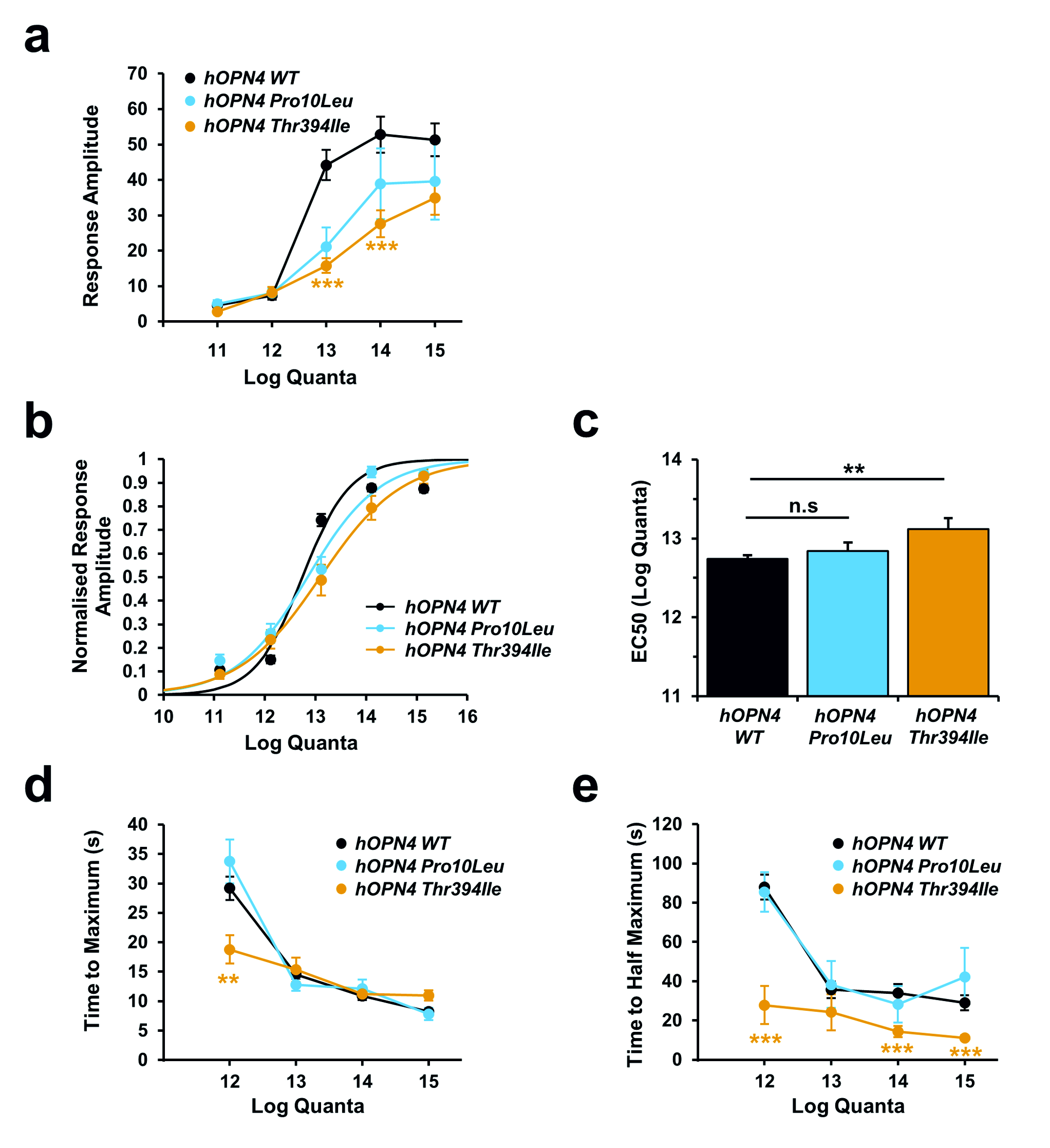

Supplement: Supplementary Data [file ddy150_supp.zip › ddy150-suppl_data/FigS5.tif]

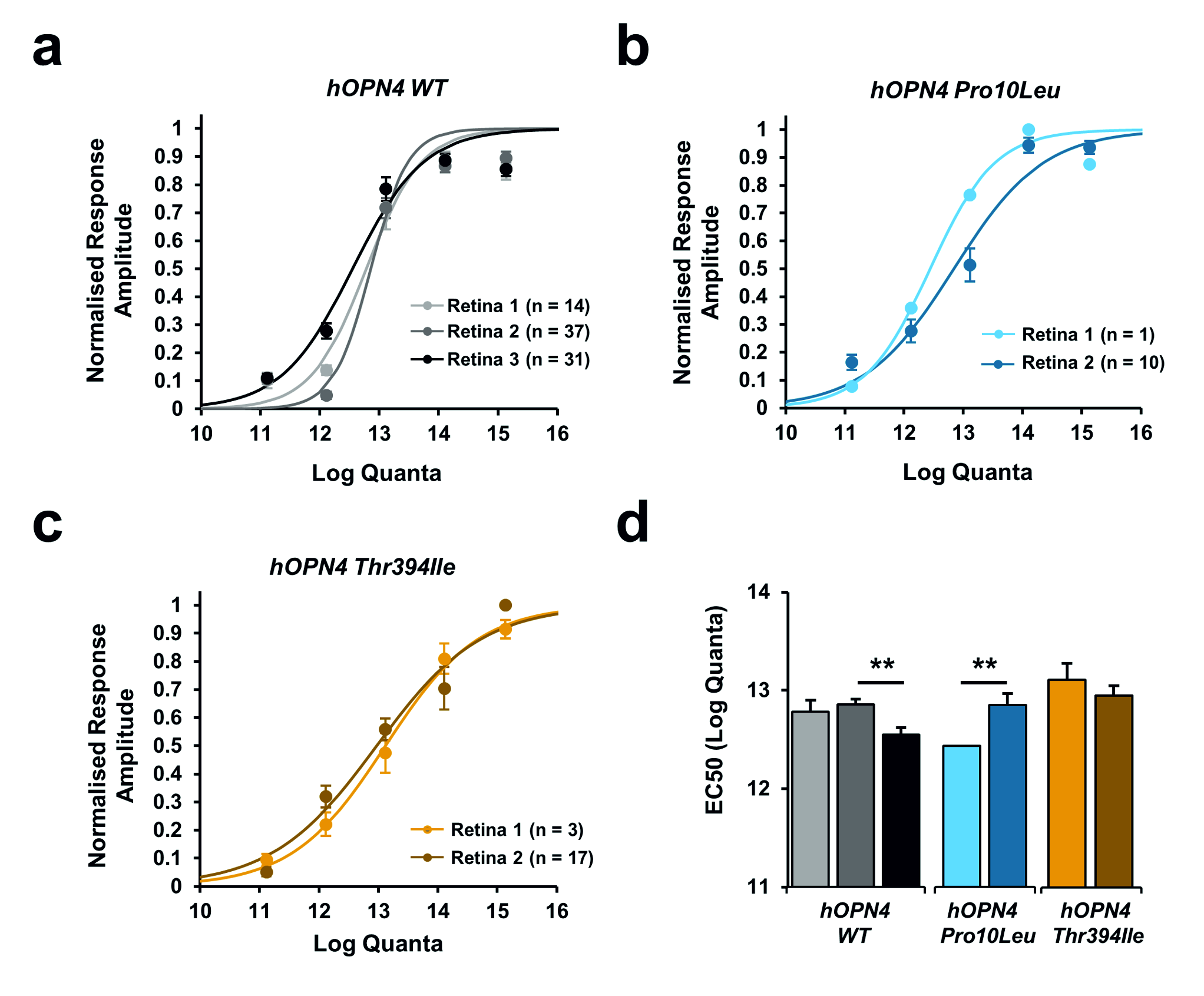

Supplement: Supplementary Data [file ddy150_supp.zip › ddy150-suppl_data/FigS6.tif]

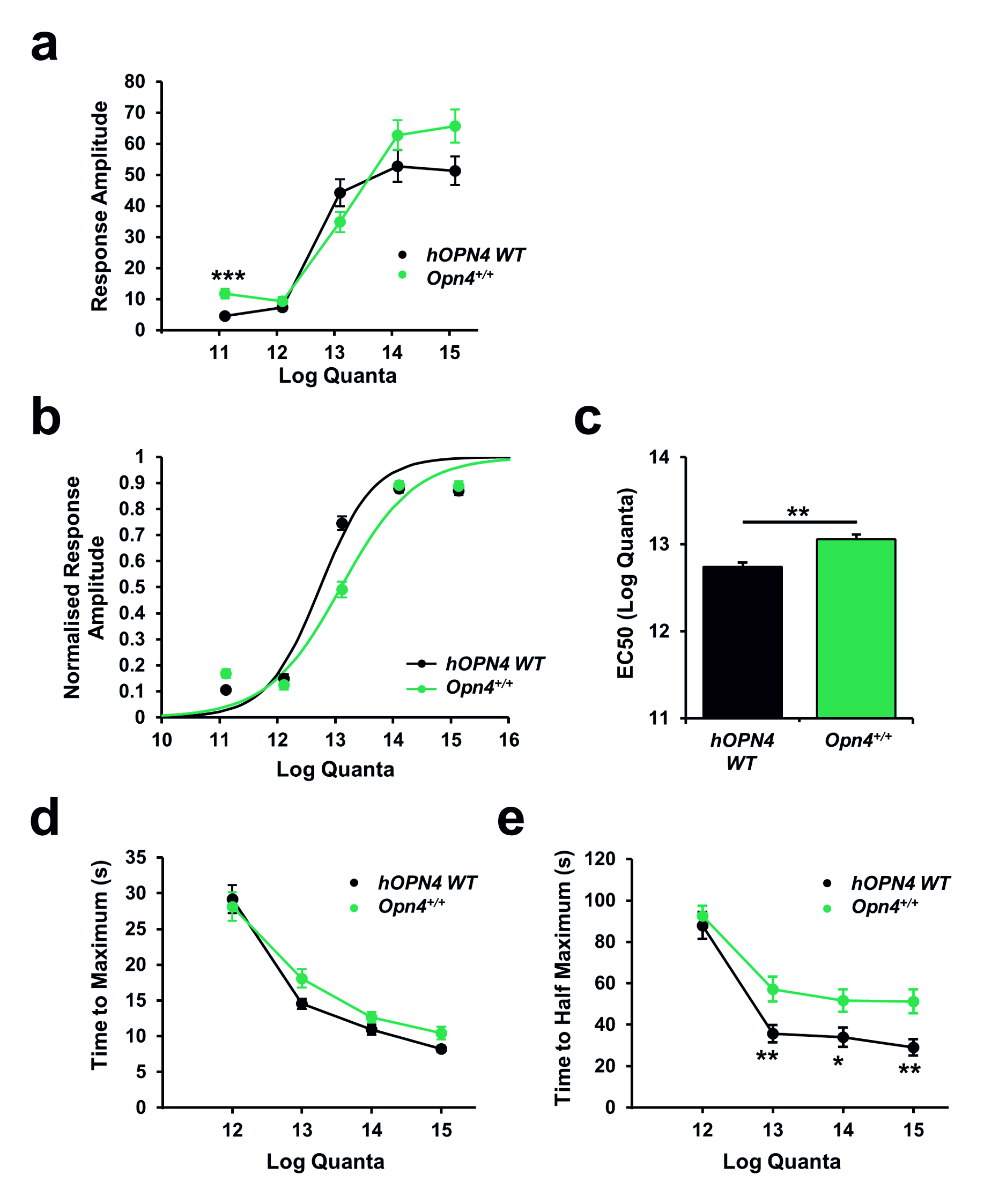

Supplement: Supplementary Data [file ddy150_supp.zip › ddy150-suppl_data/FigS7.tif]
